# Supplementary figures and images for: Breadth of CD8 T-cell mediated inhibition of replication of diverse HIV-1 transmitted-founder isolates correlates with the breadth of recognition within a comprehensive HIV-1 Gag, Nef, Env and Pol potential T-cell epitope (PTE) peptide set
Source: PLoS One. 2021 Nov 17;16(11):e0260118. doi: 10.1371/journal.pone.0260118 (PMC8598018; doi:10.1371/journal.pone.0260118)

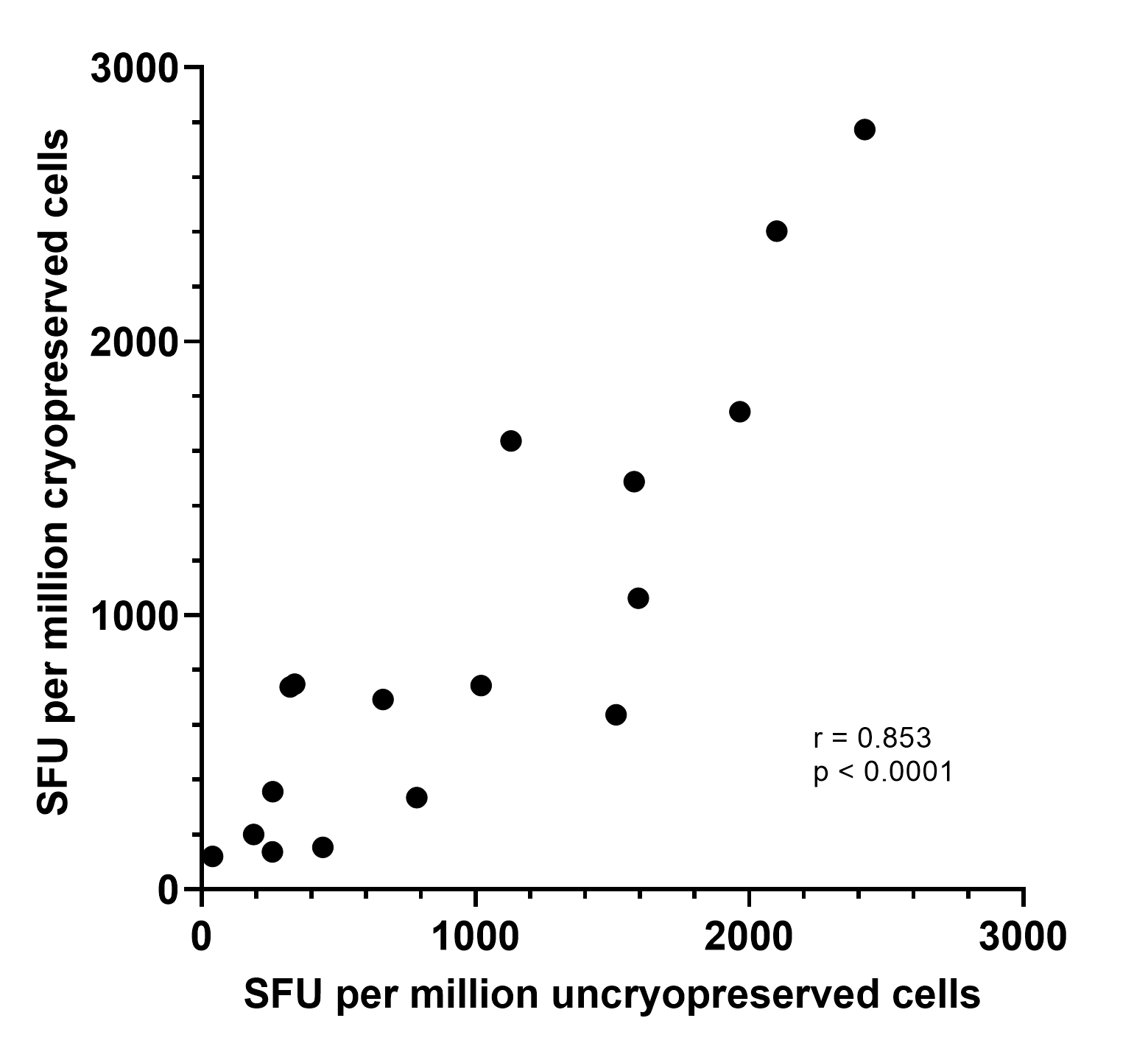

Supplement: S1 Fig — A non-parametric Spearman test was used to compute the correlation coefficients (r) between the datasets. (TIF) [file pone.0260118.s001.tif]

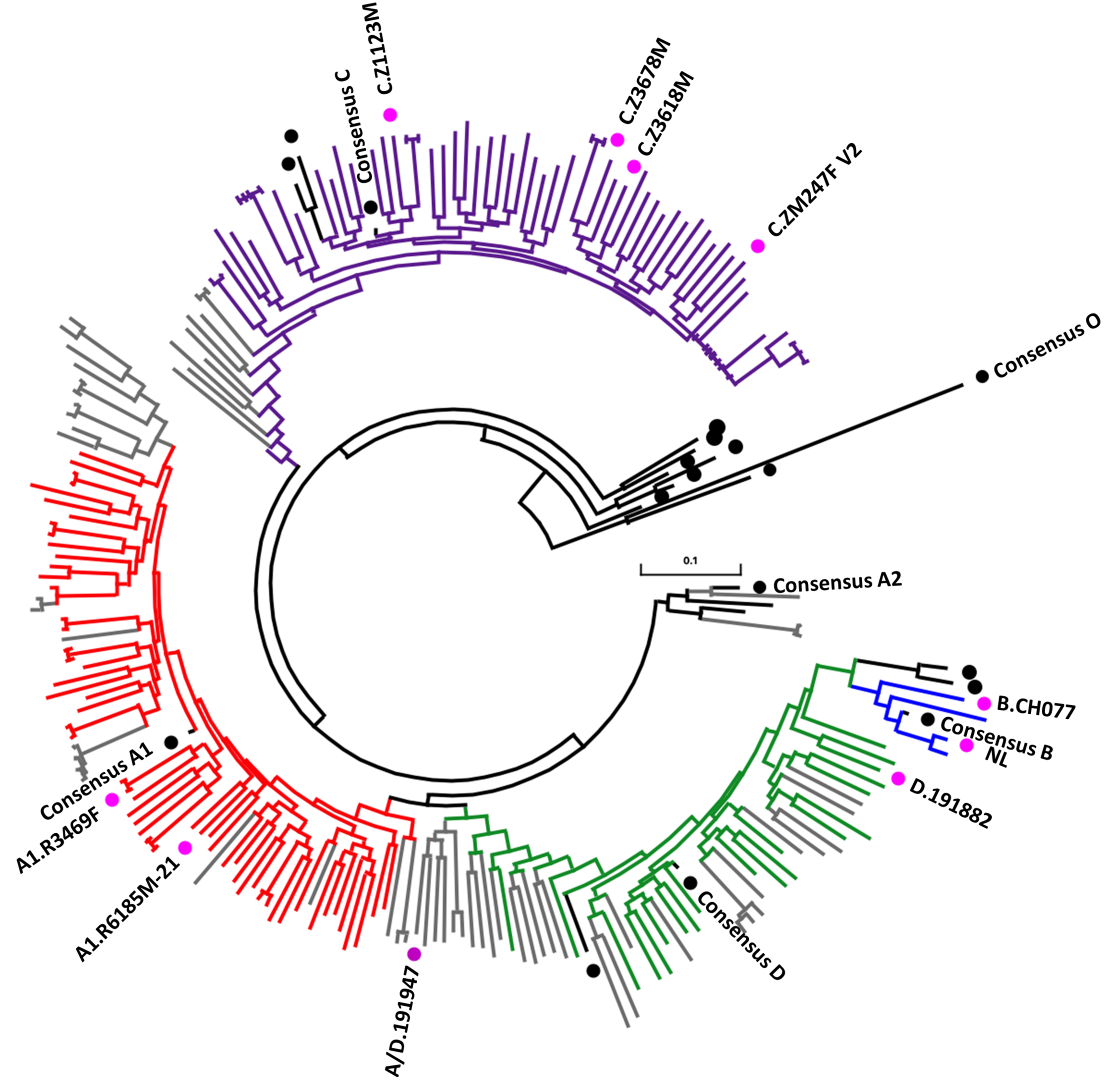

Supplement: S2 Fig — Single genome nucleotide sequences for each viral gene (gag, pol, vif, vpr, vpu, tat, rev, env, & nef) were translated to amino acids, concatenated, aligned with LANL consensus/ancestral sequences and a maximum likelihood tree generated. Transmitted/founder sequences of subtype A are shown in red, subtype B in blue, subtype C in purple, subtype D in green and unique recombinant forms in grey. Infectious molecular clones in the LucR VIA are highlighted with a pink circle. Subtype references are highlighted with a black circle. (TIF) [file pone.0260118.s002.tif]

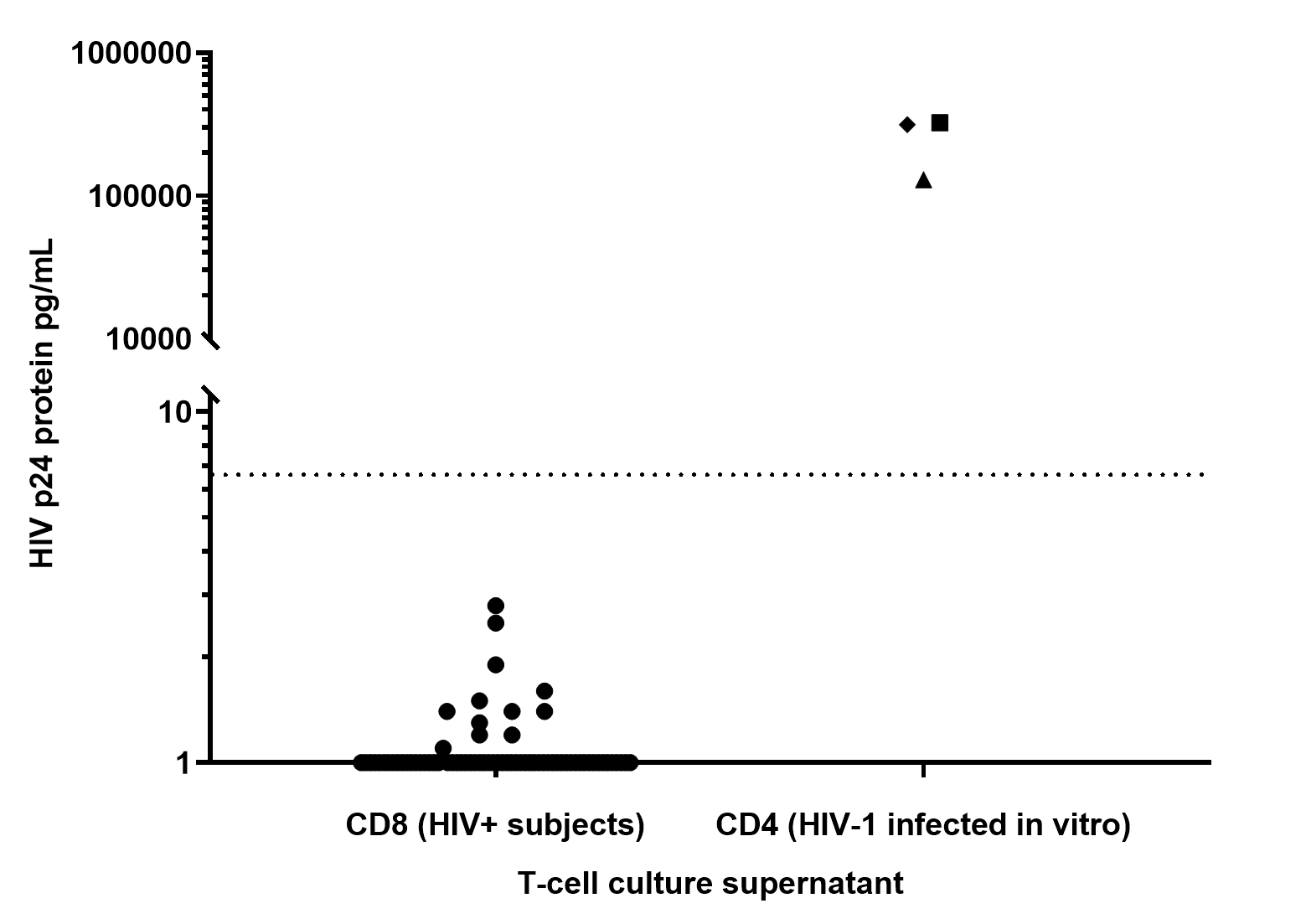

Supplement: S3 Fig — 7 day expanded CD4 T-cells from a HIV-1 uninfected subject sampled at 13 days after in vitro infection with one of three HIV-1 isolates at an MOI of 0.01: 247Fv2 (square), IIIB (triangle) and CH077 (diamond) and 10 day expanded CD8 T-cells from 71 ARV-naïve subjects living with HIV without further HIV-1 infection in vitro (circles). Dotted line represents the assay positive cut-off value (mean of replicate media only background wells OD490 + 0.05 = 5.8pg/mL) as recommended by the ELISA supplier’s instructions. (TIF) [file pone.0260118.s003.tif]
