# Supplementary material for: Breadth of CD8 T-cell mediated inhibition of replication of diverse HIV-1 transmitted-founder isolates correlates with the breadth of recognition within a comprehensive HIV-1 Gag, Nef, Env and Pol potential T-cell epitope (PTE) peptide set
Source: PLoS One. 2021 Nov 17;16(11):e0260118. doi: 10.1371/journal.pone.0260118 (PMC8598018; doi:10.1371/journal.pone.0260118)
Supplement: S1 Table — Cells were stained in 100μL staining volume at the dilution specified. (DOCX) [file pone.0260118.s004.docx]

**S1 table.** **Commercial reagents used for flow cytometry.** Cells were stained in 100μL staining volume at the dilution specified.

| **Antibody Specificity** | **Fluorochrome** | **Antibody clone** | **Vendor** | **Catalog number** | **Dilution factor** |
| --- | --- | --- | --- | --- | --- |
| CD3 | BUV790 | SK7 | Becton Dickinson, USA | 565511 | 40 |
| CD4 | BB790 | SK3 | Becton Dickinson, USA | 624296 | 160 |
| CD8 | BUV496 | RPA-T8 | Becton Dickinson, USA | 564804 | 80 |
| CD19 | BUV615 | HIB19 | Becton Dickinson, USA | 624297 | 80 |
| IFNγ | APC | B27 | Becton Dickinson, USA | 554702 | 160 |
| Dead cells | APC-Cy7 | Fixable near-IR dead cell stain | ThermoFisher, UK | L34975 | 800 |
