# Supplementary material for: Breadth of CD8 T-cell mediated inhibition of replication of diverse HIV-1 transmitted-founder isolates correlates with the breadth of recognition within a comprehensive HIV-1 Gag, Nef, Env and Pol potential T-cell epitope (PTE) peptide set
Source: PLoS One. 2021 Nov 17;16(11):e0260118. doi: 10.1371/journal.pone.0260118 (PMC8598018; doi:10.1371/journal.pone.0260118)
Supplement: S2 Table — (DOCX) [file pone.0260118.s005.docx]

**S2 table. PTE peptides recognised by CD8 T-cells from study subjects and IFNγ ELISpot response magnitudes**

|  |  |  |  |  |  |
| --- | --- | --- | --- | --- | --- |
| **Study subject** | **HIV-1 Protein** | **HXB2^a^ location** | **Peptide number^b^** | **Peptide amino acid sequence** | **SFU^c^** |
|  |  |  |  |  |  |
| 1 | Nef | 76 | 32 | LRPMTYKGALDLSHF | 250 |
|  |  | 99 | 18 | GLIYSKKRQEILDLW | 48 |
|  |  | 103 | 64 | SRRRQEILDLWVYNT | 2130 |
|  |  | 105 | 7 | KRQEILDLWVYHTQG | 2188 |
|  |  |  |  |  |  |
|  | Pol | 406 | 10 | SWTVNDIQKLVGKLN | 318 |
|  |  |  |  |  |  |
|  |  |  |  |  |  |
| 2 | Gag | 174 | 8 | ALSEGATPQDLNTML | 2110 |
|  |  | 181 | 18 | PQDLNTMLNTVGGHQ | 1580 |
|  |  | 380 | 275 | KGNFRGQKRIKCFNC | 280 |
|  |  | 397 | 212 | KEGHLARNCKAPRKK | 700 |
|  |  | 398 | 50 | EGHLARNCRAPRKKG | 278 |
|  |  |  |  |  |  |
|  | Nef | 22 | 55 | RAEPAADGVGAVSRD | 455 |
|  |  | 84 | 78 | AFDLSHFLKEKGGLD | 310 |
|  |  | 99 | 18 | GLIYSKKRQEILDLW | 55 |
|  |  | 102 | 47 | YSQKRQDILDLWVYH | 140 |
|  |  | 103 | 64 | SRRRQEILDLWVYNT | 38 |
|  |  | 105 | 17 | KRQDILDLWVYNTQG | 130 |
|  |  |  |  |  |  |
|  | Pol | 261 | 9 | VTVLDVGDAYFSVPL | 930 |
|  |  |  |  |  |  |
|  | Env | 506 | 411 | VEREKRAVGLGALFL | 308 |
|  |  | 787 | 358 | RRGWEALKYLGNLVQ | 225 |
|  |  |  |  |  |  |
|  |  |  |  |  |  |
| 3 | Gag | 16 | 26 | WEKIRLRPGGKKKYR | 98 |
|  |  |  |  |  |  |
|  | Nef | 78 | 50 | PMTYKGAFDLSHFLK | 1230 |
|  |  | 80 | 37 | TYKAAVDLSHFLKEK | 200 |
|  |  | 80 | 49 | TFKGALDLSHFLKEK | 770 |
|  |  | 81 | 29 | YKGAFDLSFFLKEKG | 1415 |
|  |  | 84 | 43 | AFDLGFFLKEKGGLE | 863 |
|  |  | 84 | 78 | AFDLSHFLKEKGGLD | 2390 |
|  |  | 176 | 113 | EREVLEWRFDSRLAF | 265 |
|  |  | 176 | 121 | EKEVLVWKFDSRLAF | 325 |
|  |  | 177 | 72 | REVLMWKFDSRLALK | 103 |
|  |  | 179 | 94 | VLVWRFDSRLAFHHV | 135 |
|  |  | 181 | 126 | MWKFDSRLAFHHVAR | 115 |
|  |  | 182 | 75 | WKFDSRLAFHHMARE | 85 |
|  |  | 183 | 85 | KFDSRLALKHRAQEL | 110 |
|  |  |  |  |  |  |
|  | Pol | 98 | 21 | WKPKMIGGIGGFIKV | 463 |
|  |  | 310 | 141 | GSPAIFQSSMTKILD | 150 |
|  |  | 313 | 72 | AIFQSSMTKILEPFR | 245 |
|  |  | 313 | 358 | AIFQSSMTRILEPFR | 160 |
|  |  | 711 | 46 | IRKVLFLDGIDKAQE | 83 |
|  |  | 738 | 393 | ASEFNLPPIVAKEII | 388 |
|  |  | 741 | 105 | FNLPPIVAKEIVASC | 1225 |
|  |  | 741 | 113 | FNLPPVVAKEIVASC | 515 |
|  |  | 846 | 135 | WWAGIQQEFGIPYNP | 970 |
|  |  | 846 | 378 | WWANVTQEFGIPYNP | 288 |
|  |  | 895 | 236 | VLIHNFKRKGGIGGY | 1233 |
|  |  | 919 | 314 | IATDIQTRELQKQII | 728 |
|  |  | 972 | 71 | IKVVPRRKAKIIRDY | 1020 |
|  |  | 975 | 147 | IPRRKAKIIRDYGKQ | 525 |
|  |  |  |  |  |  |
|  |  |  |  |  |  |
| 4 | Gag | 16 | 26 | WEKIRLRPGGKKKYR | 355 |
|  |  | 71 | 120 | GSEELRSLYNTVATL | 265 |
|  |  | 77 | 54 | SLYNTVATLYCVHQR | 458 |
|  |  | 142 | 127 | MVHQALSPRTLNAWV | 1670 |
|  |  | 142 | 214 | VVHQPISPRTLNAWV | 613 |
|  |  | 179 | 77 | ATPQDLNMMLNIVGG | 965 |
|  |  | 198 | 47 | MEMLKDTINEEAAEW | 1965 |
|  |  | 198 | 80 | MHMLKETINEEAAEW | 1945 |
|  |  | 204 | 16 | TINEEAAEWDRLHPV | 1615 |
|  |  | 235 | 21 | DIAGTTSTLQEQIGW | 603 |
|  |  | 239 | 89 | TTSTLQEQIAWMTSN | 1440 |
|  |  | 241 | 78 | STLQEQIGWMTSNPP | 405 |
|  |  | 241 | 246 | STLQEQIQWMTSNPP | 1323 |
|  |  | 304 | 67 | LRAEQATQEVKNWMT | 143 |
|  |  | 359 | 234 | KAKVLAEAMSQVQQT | 65 |
|  |  | 361 | 158 | RVLAEAMSQATNAAT | 118 |
|  |  | 362 | 266 | VLAEAMSQANNTNIM | 465 |
|  |  | 362 | 307 | VLAEAMSQAQQTNIM | 435 |
|  |  | 362 | 320 | VLAEAMSQASGAAAA | 460 |
|  |  |  |  |  |  |
|  | Nef | 129 | 10 | GPGVRYPLTFGWCFK | 300 |
|  |  | 129 | 31 | GPGTRFPLTFGWCFK | 280 |
|  |  | 183 | 85 | KFDSRLALKHRAQEL | 73 |
|  |  |  |  |  |  |
|  | Pol | 393 | 168 | KWTVQPIQLPEKDSW | 188 |
|  |  | 509 | 266 | YARKRSAHTNDVKQL | 975 |
|  |  | 512 | 330 | KRSAHTNDVRQLTEV | 73 |
|  |  | 589 | 331 | IAGVETFYVDGAASR | 553 |
|  |  | 591 | 52 | GAETFYVDGAANRET | 493 |
|  |  | 801 | 480 | AEVISAETGQETAYY | 925 |
|  |  | 806 | 107 | AETGQETAYFLLKLA | 765 |
|  |  | 807 | 121 | ETGQETAYFILKLAG | 635 |
|  |  |  |  |  |  |
|  | Env | 87 | 273 | ENVTEEFNMWKNNMV | 1578 |
|  |  | 837 | 176 | GRAILNIPRRIRQGL | 1753 |
|  |  | 838 | 92 | RAILHIPRRIRQGFE | 958 |
|  |  |  |  |  |  |
|  |  |  |  |  |  |
| 5 | Gag | 76 | 220 | KSLYNTVAVLYCVHQ | 110 |
|  |  | 198 | 47 | MEMLKDTINEEAAEW | 695 |
|  |  | 198 | 80 | MHMLKETINEEAAEW | 570 |
|  |  | 204 | 16 | TINEEAAEWDRLHPV | 440 |
|  |  | 259 | 7 | GEIYKRWIILGLNKI | 970 |
|  |  | 265 | 1 | WIILGLNKIVRMYSP | 735 |
|  |  | 304 | 82 | LRAEQATQDVKNWMT | 280 |
|  |  | 348 | 27 | TACQGVGGPGHKARV | 1855 |
|  |  | 348 | 45 | TACQGVGGPSHKARV | 850 |
|  |  |  |  |  |  |
|  | Nef | 76 | 32 | LRPMTYKGALDLSHF | 400 |
|  |  | 78 | 50 | PMTYKGAFDLSHFLK | 1305 |
|  |  | 78 | 102 | PMTFKAAFDLSFFLK | 1130 |
|  |  | 81 | 29 | YKGAFDLSFFLKEKG | 2205 |
|  |  | 84 | 43 | AFDLGFFLKEKGGLE | 200 |
|  |  | 99 | 18 | GLIYSKKRQEILDLW | 345 |
|  |  | 102 | 47 | YSQKRQDILDLWVYH | 1900 |
|  |  | 103 | 64 | SRRRQEILDLWVYNT | 2120 |
|  |  | 105 | 7 | KRQEILDLWVYHTQG | 2400 |
|  |  | 105 | 17 | KRQDILDLWVYNTQG | 1865 |
|  |  | 113 | 8 | WVYHTQGYFPDWQNY | 820 |
|  |  | 115 | 13 | YHTQGFFPDWQNYTP | 160 |
|  |  | 118 | 2 | GYFPDWQNYTPGPGV | 735 |
|  |  | 129 | 27 | GPGIRYPLTFGWCYK | 335 |
|  |  |  |  |  |  |
|  | Pol | 110 | 130 | IKVRQYDQILIEICG | 75 |
|  |  | 130 | 37 | TVLVGPTPVNIIGRN | 175 |
|  |  | 169 | 39 | PGMDGPKVKQWPLTE | 270 |
|  |  | 192 | 102 | ICTEMEKEGKISKIG | 380 |
|  |  | 204 | 57 | KIGPENPYNTPVFAI | 610 |
|  |  | 319 | 144 | MTKILEPFRKQNPDI | 205 |
|  |  | 335 | 8 | IYQYMDDLYVGSDLE | 50 |
|  |  | 363 | 94 | HLLKWGFTTPDKKHQ | 710 |
|  |  | 400 | 91 | VLPEKDSWTVNDIQK | 240 |
|  |  | 406 | 10 | SWTVNDIQKLVGKLN | 1125 |
|  |  | 418 | 103 | KLNWASQIYPGIKVR | 390 |
|  |  | 423 | 231 | SQIYPGIKVKQLCKC | 325 |
|  |  | 423 | 348 | SQIYPGIKVRQLCKC | 375 |
|  |  | 440 | 146 | GAKALTDIVPLTEEA | 200 |
|  |  | 452 | 32 | EEAELELAENREILK | 70 |
|  |  | 484 | 132 | IQKQGQDQWTYQIYQ | 45 |
|  |  | 484 | 183 | IQKQGQGQWTYQIYQ | 330 |
|  |  | 490 | 56 | DQWTYQIYQEPFKNL | 95 |
|  |  | 529 | 449 | KIALESIVIWGKTPK | 270 |
|  |  | 647 | 7 | EVNIVTDSQYALGII | 65 |
|  |  | 653 | 40 | DSQYALGIIQAQPDK | 1995 |
|  |  | 696 | 51 | GIGGNEQVDKLVSSG | 260 |
|  |  | 760 | 67 | LKGEAMHGQVDCSPG | 230 |
|  |  | 766 | 25 | HGQVDCSPGIWQLDC | 50 |
|  |  | 782 | 98 | HLEGKVILVAVHVAS | 170 |
|  |  | 784 | 148 | EGKIILVAVHVASRY | 60 |
|  |  | 838 | 327 | STAVKAACWWANVTQ | 85 |
|  |  | 876 | 86 | LIGQVRDQAEHLKTA | 210 |
|  |  | 894 | 22 | AVFIHNFKRKGGIGG | 1315 |
|  |  |  |  |  |  |
|  | Env | 33 | 8 | NLWVTVYYGVPVWKE | 170 |
|  |  | 58 | 309 | AKAYEREVHNVWATH | 175 |
|  |  | 373 | 55 | THSFNCRGEFFYCNT | 70 |
|  |  | 385 | 398 | CNTSGLFNSTWNDTG | 980 |
|  |  | 601 | 128 | KLICTTNVPWNSTWS | 60 |
|  |  | 656 | 153 | NEKDLLALDSWKNLW | 160 |
|  |  | 738 | 321 | GEQDKDRSIRLVNGF | 115 |
|  |  | 741 | 210 | DRDRSVRLVSGFLAL | 115 |
|  |  | 771 | 135 | LRDFILIAARTVELL | 425 |
|  |  | 777 | 266 | IAARTVELLGRSSLK | 450 |
|  |  | 780 | 76 | RIVELLGRRGWEALK | 95 |
|  |  | 840 | 177 | ILHIPTRIRQGLERA | 250 |
|  |  | 842 | 56 | HIPRRIRQGLERALL | 400 |
|  |  |  |  |  |  |
|  |  |  |  |  |  |
| 6 | Gag | 174 | 8 | ALSEGATPQDLNTML | 1601 |
|  |  | 179 | 77 | ATPQDLNMMLNIVGG | 213 |
|  |  | 181 | 18 | PQDLNTMLNTVGGHQ | 1341 |
|  |  | 253 | 31 | NPPIPVGEIYKRWII | 187 |
|  |  | 255 | 53 | PIPVGDIYKRWIILG | 520 |
|  |  | 255 | 223 | PVPVGEIYKRWIVLG | 240 |
|  |  | 259 | 7 | GEIYKRWIILGLNKI | 340 |
|  |  |  |  |  |  |
|  | Nef | 6 | 66 | SKSSIVGWPEVRERM | 113 |
|  |  | 7 | 59 | KSSIVGWPAVRERIR | 113 |
|  |  | 10 | 120 | GVGWPTVRERMRRAE | 145 |
|  |  | 11 | 108 | VGWPAVRERMRRTEP | 138 |
|  |  | 12 | 122 | GWPEVRERMRRAPAA | 91 |
|  |  | 13 | 62 | WPAVRERMRRAEPAA | 774 |
|  |  | 66 | 1 | VGFPVRPQVPLRPMT | 587 |
|  |  | 68 | 51 | FPVKPQVPLRPMTFK | 47 |
|  |  | 81 | 29 | YKGAFDLSFFLKEKG | 1501 |
|  |  | 84 | 43 | AFDLGFFLKEKGGLE | 580 |
|  |  | 86 | 5 | DLSHFLKEKGGLEGL | 213 |
|  |  | 87 | 11 | LSFFLKEKGGLDGLI | 767 |
|  |  | 102 | 47 | YSQKRQDILDLWVYH | 60 |
|  |  | 103 | 87 | SQRRQDILDLWIYHT | 38 |
|  |  | 105 | 17 | KRQDILDLWVYNTQG | 60 |
|  |  | 129 | 10 | GPGVRYPLTFGWCFK | 153 |
|  |  | 129 | 27 | GPGIRYPLTFGWCYK | 107 |
|  |  | 129 | 31 | GPGTRFPLTFGWCFK | 120 |
|  |  | 185 | 68 | DSSLARRHMARELHP | 73 |
|  |  |  |  |  |  |
|  | Pol | 589 | 331 | IAGVETFYVDGAASR | 1772 |
|  |  | 591 | 52 | GAETFYVDGAANRET | 1079 |
|  |  | 606 | 82 | KLGKAGYVTDRGRQK | 171 |
|  |  | 612 | 134 | YVTDRGRQKVVSLTE | 131 |
|  |  | 612 | 329 | YVTDRGRQKIVSLTE | 211 |
|  |  | 828 | 85 | IHTDNGSNFTSAAVK | 71 |
|  |  |  |  |  |  |
|  | Env | 320 | 64 | GDIIGDIRQAHCNIS | 133 |
|  |  | 368 | 201 | DLEIVMHSFNCGGEF | 247 |
|  |  | 583 | 25 | VERYLKDQQLLGIWG | 894 |
|  |  | 583 | 399 | VEKYLKDQQLLGLWG | 674 |
|  |  | 674 | 269 | DISNWLWYIRIFIII | 554 |
|  |  | 792 | 148 | ALKYLGSLVQYWGLE | 454 |
|  |  |  |  |  |  |
|  |  |  |  |  |  |
| 7 | Gag | 140 | 42 | GQMVHQAISPRTLNA | 65 |
|  |  |  |  |  |  |
|  | Nef | 103 | 64 | SRRRQEILDLWVYNT | 270 |
|  |  | 105 | 7 | KRQEILDLWVYHTQG | 130 |
|  |  |  |  |  |  |
|  | Pol | 285 | 295 | FTIPSINNETPGVRY | 335 |
|  |  | 286 | 79 | TIPSINNETPGIRYQ | 400 |
|  |  | 287 | 264 | IPSTNNETPGIRYCY | 495 |
|  |  | 287 | 350 | IPSVNNETPGIRYIY | 530 |
|  |  | 292 | 36 | NETPGIRYQYNVLPQ | 520 |
|  |  | 324 | 335 | EPFRAQNPEIVIYQY | 455 |
|  |  | 324 | 459 | EPFRKQNPEMVIYQY | 230 |
|  |  | 329 | 110 | KNPEIVIYQYMDDLY | 250 |
|  |  | 329 | 354 | KNPEIIIYQYMDDLY | 268 |
|  |  | 357 | 278 | IEELREHLLRWGFTT | 115 |
|  |  | 357 | 287 | VEELREHLLKWGFTT | 200 |
|  |  | 627 | 324 | TANQKTELQAIQLAL | 63 |
|  |  | 807 | 121 | ETGQETAYFILKLAG | 430 |
|  |  | 807 | 239 | ETGQETAYYILKLAG | 790 |
|  |  | 823 | 214 | WPVKVVHTDNGSNFT | 125 |
|  |  | 824 | 290 | PVKTIHTDNGSNFIS | 1935 |
|  |  | 825 | 194 | VKVIHTDNGSNFTST | 1160 |
|  |  | 828 | 85 | IHTDNGSNFTSAAVK | 2030 |
|  |  | 828 | 425 | IHADNGSNFTSTTVK | 1560 |
|  |  | 846 | 135 | WWAGIQQEFGIPYNP | 75 |
|  |  | 888 | 5 | KTAVQMAVFIHNFKR | 735 |
|  |  | 894 | 22 | AVFIHNFKRKGGIGG | 75 |
|  |  | 895 | 236 | VLIHNFKRKGGIGGY | 1490 |
|  |  | 972 | 71 | IKVVPRRKAKIIRDY | 265 |
|  |  | 975 | 147 | IPRRKAKIIRDYGKQ | 55 |
|  |  | 975 | 288 | VPRRKVKIIRDYGKQ | 45 |
|  |  |  |  |  |  |
|  | Env | 842 | 217 | NTPRRIRQGFEAALL | 50 |
|  |  |  |  |  |  |
|  |  |  |  |  |  |
| 8 | Gag | 25 | 175 | GKKHYMLKHLVWASR | 1041 |
|  |  | 25 | 303 | GKKHYMIKHLVWASR | 1525 |
|  |  | 32 | 20 | KHLVWASRELERFAL | 73 |
|  |  | 37 | 68 | ASRELERFAVNPGLL | 327 |
|  |  | 44 | 58 | FALNPGLLETSEGCR | 280 |
|  |  | 45 | 167 | ALNPGLLETAEGCQQ | 714 |
|  |  | 61 | 189 | LGQLQPALQTGSEEL | 527 |
|  |  | 62 | 317 | GQLHPSLQTGSEELK | 85 |
|  |  | 64 | 118 | LQPSLQTGSEELRSL | 206 |
|  |  | 67 | 182 | ALKTGTEELRSLYNT | 133 |
|  |  | 67 | 219 | ALQTGTEELKSLYNT | 1094 |
|  |  | 67 | 249 | ALQTGSEELRSLFNT | 425 |
|  |  | 71 | 120 | GSEELRSLYNTVATL | 514 |
|  |  | 71 | 123 | GSEELKSLYNTVATL | 687 |
|  |  | 74 | 88 | ELRSLFNTVATLYCV | 367 |
|  |  | 76 | 220 | KSLYNTVAVLYCVHQ | 414 |
|  |  | 76 | 289 | KSLFNTVATLYCVHA | 545 |
|  |  | 77 | 54 | SLYNTVATLYCVHQR | 907 |
|  |  | 154 | 75 | NAVKVVEEKAFSPEV | 153 |
|  |  | 198 | 47 | MEMLKDTINEEAAEW | 793 |
|  |  | 198 | 80 | MHMLKETINEEAAEW | 807 |
|  |  | 204 | 16 | TINEEAAEWDRLHPV | 433 |
|  |  | 251 | 185 | TSNPPVPVGDIYKRW | 87 |
|  |  | 255 | 53 | PIPVGDIYKRWIILG | 306 |
|  |  | 304 | 82 | LRAEQATQDVKNWMT | 1080 |
|  |  | 474 | 284 | PKQEQKDKELYPLAS | 38 |
|  |  | 485 | 90 | PLTSLKSLFGSDPLS | 460 |
|  |  | 486 | 208 | LTSLRSLFGSDPLSQ | 1294 |
|  |  | 486 | 217 | LTSLRSLFGNDPLSQ | 527 |
|  |  |  |  |  |  |
|  | Nef | 81 | 29 | YKGAFDLSFFLKEKG | 285 |
|  |  | 84 | 43 | AFDLGFFLKEKGGLE | 238 |
|  |  | 129 | 10 | GPGVRYPLTFGWCFK | 2212 |
|  |  | 129 | 27 | GPGIRYPLTFGWCYK | 1612 |
|  |  | 129 | 31 | GPGTRFPLTFGWCFK | 2146 |
|  |  |  |  |  |  |
|  | Pol | 7 | 260 | AFPQGEAREFPSEQT | 144 |
|  |  | 112 | 285 | VKQYDQILIEICGKK | 44 |
|  |  | 393 | 168 | KWTVQPIQLPEKDSW | 1458 |
|  |  | 412 | 4 | IQKLVGKLNWASQIY | 1044 |
|  |  | 418 | 103 | KLNWASQIYPGIKVR | 190 |
|  |  | 418 | 136 | KLNWASQIYAGIKVK | 1178 |
|  |  | 527 | 304 | VQKIAMESIVIWGKT | 1584 |
|  |  | 527 | 477 | VQKVVMESIVIWGKA | 317 |
|  |  | 612 | 329 | YVTDRGRQKIVSLTE | 97 |
|  |  | 928 | 151 | LQKQIIKIQNFRVYY | 2291 |
|  |  | 933 | 301 | TKIQNFRVYYRDNRD | 1751 |
|  |  | 934 | 42 | KIQNFRVYYRDSRDP | 2332 |
|  |  |  |  |  |  |
|  | Env | 604 | 175 | CTTAVPWNSSWSNRS | 60 |
|  |  | 838 | 428 | RAILHIPTRIRQGFE | 106 |
|  |  |  |  |  |  |
|  |  |  |  |  |  |
| 9 | Gag | 31 | 245 | IKHLVWASRELDRFA | 485 |
|  |  | 32 | 20 | KHLVWASRELERFAL | 1405 |
|  |  | 56 | 211 | GCKQIMKQLQPALQT | 85 |
|  |  | 57 | 279 | CKQIIKQLQPALQTG | 53 |
|  |  | 71 | 120 | GSEELRSLYNTVATL | 45 |
|  |  | 74 | 88 | ELRSLFNTVATLYCV | 523 |
|  |  | 76 | 220 | KSLYNTVAVLYCVHQ | 273 |
|  |  | 77 | 54 | SLYNTVATLYCVHQR | 113 |
|  |  | 89 | 289 | HQRIDIKDTKEALEK | 253 |
|  |  | 140 | 42 | GQMVHQAISPRTLNA | 1585 |
|  |  | 142 | 127 | MVHQALSPRTLNAWV | 1898 |
|  |  | 142 | 214 | VVHQPISPRTLNAWV | 2660 |
|  |  | 142 | 290 | MTHQSMSPRTLNAWV | 995 |
|  |  | 146 | 190 | AISPRTLNAWVKAIE | 1948 |
|  |  | 147 | 17 | ISPRTLNAWVKVIEE | 1455 |
|  |  | 160 | 14 | EEKAFSPEVIPMFSA | 1363 |
|  |  | 160 | 192 | EEKGFNPEVIPMFSA | 110 |
|  |  | 161 | 111 | EKGFSPEVIPMFTAL | 1665 |
|  |  | 292 | 28 | PFRDYVDRFFKTLRA | 965 |
|  |  | 292 | 48 | PFRDYVDRFYKTLRA | 228 |
|  |  | 304 | 82 | LRAEQATQDVKNWMT | 988 |
|  |  | 474 | 195 | PKQEPKDREPLTSLK | 95 |
|  |  |  |  |  |  |
|  | Nef | 66 | 1 | VGFPVRPQVPLRPMT | 140 |
|  |  | 68 | 51 | FPVKPQVPLRPMTFK | 93 |
|  |  | 76 | 32 | LRPMTYKGALDLSHF | 78 |
|  |  | 76 | 46 | LRPMTYKAAFDLSFF | 393 |
|  |  | 78 | 50 | PMTYKGAFDLSHFLK | 115 |
|  |  | 78 | 102 | PMTFKAAFDLSFFLK | 98 |
|  |  | 81 | 29 | YKGAFDLSFFLKEKG | 358 |
|  |  | 111 | 33 | DLWVYHTQGFFPDWH | 498 |
|  |  | 111 | 44 | DLWVYNTQGFFPDWQ | 305 |
|  |  | 113 | 8 | WVYHTQGYFPDWQNY | 975 |
|  |  | 115 | 13 | YHTQGFFPDWQNYTP | 2273 |
|  |  | 118 | 2 | GYFPDWQNYTPGPGV | 228 |
|  |  | 123 | 14 | WQNYTPGPGVRYPLT | 405 |
|  |  | 123 | 16 | WQNYTPGPGIRYPLT | 190 |
|  |  |  |  |  |  |
|  | Pol | 436 | 367 | RLLRGTKALTDIVPL | 168 |
|  |  | 438 | 387 | LRGAKALTDIVTLTE | 318 |
|  |  | 440 | 146 | GAKALTDIVPLTEEA | 128 |
|  |  | 527 | 167 | VQKIATESIVIWGKT | 720 |
|  |  | 527 | 304 | VQKIAMESIVIWGKT | 310 |
|  |  | 529 | 449 | KIALESIVIWGKTPK | 395 |
|  |  | 533 | 84 | ESIVIWGKTPKFRLP | 153 |
|  |  | 558 | 55 | TEYWQATWIPEWEFV | 78 |
|  |  | 612 | 329 | YVTDRGRQKIVSLTE | 210 |
|  |  | 738 | 393 | ASEFNLPPIVAKEII | 1475 |
|  |  | 741 | 105 | FNLPPIVAKEIVASC | 1473 |
|  |  | 741 | 113 | FNLPPVVAKEIVASC | 160 |
|  |  | 838 | 327 | STAVKAACWWANVTQ | 165 |
|  |  | 888 | 5 | KTAVQMAVFIHNFKR | 1215 |
|  |  | 928 | 151 | LQKQIIKIQNFRVYY | 1880 |
|  |  | 933 | 301 | TKIQNFRVYYRDNRD | 1390 |
|  |  | 934 | 42 | KIQNFRVYYRDSRDP | 1830 |
|  |  |  |  |  |  |
|  | Env | 55 | 419 | ASDARAYDTEVHNVW | 1553 |
|  |  | 633 | 380 | REISNYTNTIYRLLE | 353 |
|  |  | 634 | 401 | EISNYTDTIYRLLEV | 293 |
|  |  | 738 | 321 | GEQDKDRSIRLVNGF | 253 |
|  |  | 804 | 184 | GLELKKSAISLLDTI | 143 |
|  |  |  |  |  |  |
| 10 |  |  |  |  |  |
|  | Gag | 45 | 167 | ALNPGLLETAEGCQQ | 85 |
|  |  | 83 | 153 | ATLYCVHQRIEVKDT | 215 |
|  |  | 84 | 312 | TLYCVHQRIDIKDTK | 245 |
|  |  | 131 | 188 | NYPIVQNIQGQMVHQ | 715 |
|  |  | 134 | 40 | IVQNLQGQMVHQAIS | 1015 |
|  |  | 161 | 111 | EKGFSPEVIPMFTAL | 1135 |
|  |  | 167 | 59 | EVIPMFTALSEGATP | 790 |
|  |  | 174 | 8 | ALSEGATPQDLNTML | 310 |
|  |  | 271 | 13 | NKIVRMYSPVSILDI | 105 |
|  |  |  |  |  |  |
|  | Nef | 51 | 92 | NNADCAWLQAQEEEE | 180 |
|  |  | 102 | 47 | YSQKRQDILDLWVYH | 530 |
|  |  | 103 | 64 | SRRRQEILDLWVYNT | 120 |
|  |  | 103 | 87 | SQRRQDILDLWIYHT | 255 |
|  |  | 105 | 7 | KRQEILDLWVYHTQG | 265 |
|  |  | 105 | 17 | KRQDILDLWVYNTQG | 385 |
|  |  |  |  |  |  |
|  | Pol | 55 | 69 | SFPQITLWQRPLVTV | 55 |
|  |  | 55 | 342 | SLPQITLWQRPIVTI | 70 |
|  |  | 59 | 219 | ITLWQRPLVSIKVGG | 290 |
|  |  | 136 | 112 | TPVNIIGRNMLTQIG | 1375 |
|  |  | 136 | 124 | TPVNIIGRNLLTQIG | 1390 |
|  |  | 144 | 143 | NMLTQLGCTLNFPIS | 125 |
|  |  | 157 | 216 | ISPIDTVPVKLKPGM | 255 |
|  |  | 181 | 115 | LTEEKIKALTEICTE | 295 |
|  |  | 233 | 233 | RELNRRTQDFWEVQL | 240 |
|  |  | 268 | 184 | DAYFSVPLDESFRKY | 45 |
|  |  | 273 | 152 | VPLDKDFRKYTAFTI | 360 |
|  |  | 279 | 58 | FRKYTAFTIPSINNE | 185 |
|  |  | 281 | 251 | KYTAFTIPSVNNETP | 655 |
|  |  | 308 | 238 | WKGSPAIFQCSMTKI | 40 |
|  |  | 324 | 459 | EPFRKQNPEMVIYQY | 115 |
|  |  | 357 | 287 | VEELREHLLKWGFTT | 85 |
|  |  | 357 | 398 | IEELRQHLLKWGFTT | 70 |
|  |  | 423 | 231 | SQIYPGIKVKQLCKC | 90 |
|  |  | 423 | 348 | SQIYPGIKVRQLCKC | 140 |
|  |  | 472 | 75 | VYYDPSKDLIAEIQK | 875 |
|  |  | 517 | 309 | TNDVKQLTEVVQKIA | 420 |
|  |  | 554 | 160 | ETWWTDYWQATWIPE | 450 |
|  |  | 570 | 13 | EFVNTPPLVKLWYQL | 210 |
|  |  | 603 | 140 | RETKLGKAGYVTDKG | 205 |
|  |  | 633 | 201 | ELQAIQLALQDSGSE | 555 |
|  |  | 677 | 435 | IEKLIGKDKVYLSWV | 160 |
|  |  | 678 | 221 | EELIKKEKVYLAWVP | 310 |
|  |  | 747 | 48 | VAKEIVASCDKCQLK | 220 |
|  |  | 749 | 423 | KEIVANCDKCQLKGE | 70 |
|  |  | 778 | 108 | LDCTHLEGKIILVAV | 135 |
|  |  | 800 | 29 | EAEVIPAETGQETAY | 115 |
|  |  | 807 | 121 | ETGQETAYFILKLAG | 80 |
|  |  | 807 | 239 | ETGQETAYYILKLAG | 90 |
|  |  | 818 | 197 | KLAGRWPVKTIHTDN | 50 |
|  |  | 841 | 158 | LKAACWWAGIKQEFG | 745 |
|  |  | 938 | 116 | FQVYYRDSRDPIWKG | 120 |
|  |  |  |  |  |  |
|  | Env | 204 | 102 | ACPKVTFEPIPIHYC | 235 |
|  |  | 205 | 257 | CPKVTFDPIPIHYCT | 910 |
|  |  | 206 | 93 | PKVSFDPIPIHYCAP | 1080 |
|  |  | 207 | 80 | KVSFEPIPIHYCTPA | 1050 |
|  |  | 209 | 318 | SWDPIPIHYCAPAGY | 225 |
|  |  | 278 | 238 | TNNAKTIIVHLNESV | 205 |
|  |  | 837 | 176 | GRAILNIPRRIRQGL | 315 |
|  |  | 842 | 56 | HIPRRIRQGLERALL | 110 |
|  |  |  |  |  |  |
|  |  |  |  |  |  |
| 11 | Gag | 67 | 182 | ALKTGTEELRSLYNT | 658 |
|  |  | 74 | 88 | ELRSLFNTVATLYCV | 118 |
|  |  | 76 | 289 | KSLFNTVATLYCVHA | 683 |
|  |  | 146 | 190 | AISPRTLNAWVKAIE | 158 |
|  |  | 160 | 14 | EEKAFSPEVIPMFSA | 188 |
|  |  | 160 | 192 | EEKGFNPEVIPMFSA | 803 |
|  |  | 161 | 111 | EKGFSPEVIPMFTAL | 333 |
|  |  | 235 | 21 | DIAGTTSTLQEQIGW | 48 |
|  |  |  |  |  |  |
|  | Pol | 267 | 129 | GDAYFSVPLDKDFRK | 268 |
|  |  | 269 | 377 | AYFSVPLDEGFRKYT | 265 |
|  |  | 423 | 348 | SQIYPGIKVRQLCKC | 958 |
|  |  | 426 | 156 | YPGIKVRQLCKLLRG | 1138 |
|  |  | 529 | 449 | KIALESIVIWGKTPK | 745 |
|  |  | 928 | 151 | LQKQIIKIQNFRVYY | 335 |
|  |  | 933 | 301 | TKIQNFRVYYRDNRD | 323 |
|  |  | 934 | 42 | KIQNFRVYYRDSRDP | 540 |
|  |  |  |  |  |  |
|  | Env | 295 | 305 | TCIRPNNNTRKSVRI | 515 |
|  |  | 634 | 401 | EISNYTDTIYRLLEV | 638 |
|  |  |  |  |  |  |
|  |  |  |  |  |  |
| 12 | Gag | 67 | 219 | ALQTGTEELKSLYNT | 50 |
|  |  | 142 | 214 | VVHQPISPRTLNAWV | 1545 |
|  |  | 150 | 43 | RTLNAWVKVVEEKAF | 300 |
|  |  |  |  |  |  |
|  | Nef | 68 | 51 | FPVKPQVPLRPMTFK | 830 |
|  |  | 74 | 21 | VPLRPMTYKAAVDLS | 310 |
|  |  | 81 | 29 | YKGAFDLSFFLKEKG | 110 |
|  |  | 111 | 24 | DLWVYNTQGYFPDWQ | 55 |
|  |  | 123 | 14 | WQNYTPGPGVRYPLT | 193 |
|  |  | 123 | 16 | WQNYTPGPGIRYPLT | 173 |
|  |  | 129 | 10 | GPGVRYPLTFGWCFK | 878 |
|  |  | 129 | 27 | GPGIRYPLTFGWCYK | 743 |
|  |  | 129 | 31 | GPGTRFPLTFGWCFK | 845 |
|  |  | 176 | 113 | EREVLEWRFDSRLAF | 50 |
|  |  | 176 | 121 | EKEVLVWKFDSRLAF | 88 |
|  |  |  |  |  |  |
|  | Pol | 418 | 136 | KLNWASQIYAGIKVK | 868 |
|  |  | 423 | 246 | SQIYAGIKVKQLCKP | 498 |
|  |  | 501 | 203 | YKNLKTGKYARMRGA | 105 |
|  |  | 507 | 444 | GKYARMRGAHTNDVR | 315 |
|  |  | 509 | 217 | YARMRGAHTNDVKQL | 398 |
|  |  | 546 | 258 | LPIQKETWEAWWTEY | 200 |
|  |  | 684 | 83 | EKVYLSWVPAHKGIG | 40 |
|  |  | 813 | 88 | AYFILKLAGRWPVKV | 858 |
|  |  | 813 | 334 | AYYILKLAGRWPVKI | 913 |
|  |  | 818 | 197 | KLAGRWPVKTIHTDN | 820 |
|  |  | 895 | 236 | VLIHNFKRKGGIGGY | 2203 |
|  |  | 928 | 151 | LQKQIIKIQNFRVYY | 165 |
|  |  | 972 | 71 | IKVVPRRKAKIIRDY | 2058 |
|  |  | 975 | 147 | IPRRKAKIIRDYGKQ | 1193 |
|  |  |  |  |  |  |
|  | Env | 619 | 294 | LNEIWDNMTWLQWDK | 105 |
|  |  |  |  |  |  |
|  |  |  |  |  |  |
| 13 | Gag | 22 | 110 | RPGGKKKYRLKHIVW | 38 |
|  |  | 23 | 260 | PGGNKKYKLKHIVWA | 143 |
|  |  | 26 | 79 | KKKYRLKHLVWASRE | 105 |
|  |  | 29 | 60 | YKLKHIVWASRELER | 183 |
|  |  | 31 | 245 | IKHLVWASRELDRFA | 98 |
|  |  | 40 | 163 | ELERFALNPSLLETT | 85 |
|  |  | 76 | 220 | KSLYNTVAVLYCVHQ | 793 |
|  |  | 76 | 289 | KSLFNTVATLYCVHA | 463 |
|  |  | 142 | 127 | MVHQALSPRTLNAWV | 1190 |
|  |  | 331 | 139 | KTILKALGPGATLED | 273 |
|  |  | 349 | 166 | ACQEVGGPGHKARVL | 98 |
|  |  | 354 | 76 | GGPSHKARVLAEAMG | 233 |
|  |  | 355 | 15 | GPGHKARVLAEAMSQ | 153 |
|  |  |  |  |  |  |
|  | Nef | 80 | 37 | TYKAAVDLSHFLKEK | 103 |
|  |  | 87 | 11 | LSFFLKEKGGLDGLI | 93 |
|  |  | 111 | 24 | DLWVYNTQGYFPDWQ | 90 |
|  |  | 129 | 10 | GPGVRYPLTFGWCFK | 565 |
|  |  | 129 | 27 | GPGIRYPLTFGWCYK | 315 |
|  |  | 129 | 31 | GPGTRFPLTFGWCFK | 425 |
|  |  |  |  |  |  |
|  | Pol | 5 | 299 | NLAFPQGKAREFSSE | 668 |
|  |  | 120 | 162 | IEICGHKAIGTVLVG | 103 |
|  |  | 484 | 291 | IQKQGHDQWTYQIYQ | 1180 |
|  |  | 612 | 329 | YVTDRGRQKIVSLTE | 45 |
|  |  | 928 | 151 | LQKQIIKIQNFRVYY | 858 |
|  |  | 933 | 301 | TKIQNFRVYYRDNRD | 948 |
|  |  | 934 | 42 | KIQNFRVYYRDSRDP | 898 |
|  |  |  |  |  |  |
|  | Env | 39 | 46 | YYGVPVWKEATTTLF | 1863 |
|  |  | 163 | 406 | TSIRDKVQKEYALFY | 233 |
|  |  | 789 | 228 | GWEGLKYLWNLLLYW | 370 |
|  |  | 791 | 280 | EALKYLWNLLQYWIQ | 858 |
|  |  | 791 | 438 | GALKYWWNLLQYWIQ | 143 |
|  |  | 795 | 338 | YLWNLLLYWGRELKN | 343 |
|  |  | 837 | 176 | GRAILNIPRRIRQGL | 1263 |
|  |  | 838 | 92 | RAILHIPRRIRQGFE | 498 |
|  |  | 842 | 56 | HIPRRIRQGLERALL | 698 |
|  |  | 842 | 142 | NIPRRIRQGFERALL | 1155 |
|  |  | 842 | 217 | NTPRRIRQGFEAALL | 1123 |

^a^Location of peptides' first amino acid relative to HIV-1 HXB2, ^b^PTE peptide number as supplied, ^c^Spot forming units (SFU) per million expanded CD8 T-cells
