# Supplementary material for: Breadth of CD8 T-cell mediated inhibition of replication of diverse HIV-1 transmitted-founder isolates correlates with the breadth of recognition within a comprehensive HIV-1 Gag, Nef, Env and Pol potential T-cell epitope (PTE) peptide set
Source: PLoS One. 2021 Nov 17;16(11):e0260118. doi: 10.1371/journal.pone.0260118 (PMC8598018; doi:10.1371/journal.pone.0260118)
Supplement: S3 Table — Inhibition was determined by the log10 reduction in relative light units of cultures of CD4 T-cells infected with one of ten luciferase gene engineered HIV-1 infectious molecular clones (IMC) and co-cultured with autologous CD8 T-cells compared with cultures of HIV-1 infected CD4 T-cells alone. (DOCX) [file pone.0260118.s006.docx]

| **LucR HIV-1 IMC** | **Log_10_ reduction in HIV-1 replication for study subjects and their HIV-1 clade** | | | | | | | | | | | | |
| --- | --- | --- | --- | --- | --- | --- | --- | --- | --- | --- | --- | --- | --- |
|  | **Subject**  **Clade A** | | | | | **Subject**  **Clade C** | | | | | | **Subject**  **Clade D** | |
|  | **1** | **2** | **3** | **4** | **5** | **6** | **7** | **8** | **9** | **10** | **11** | **12** | **13** |
|  |  |  |  |  |  |  |  |  |  |  |  |  |  |
| **A1.R6185M-21** | 0.88 | 0.48 | 1.84 | 1.13 | 1.25 | 2.10 | 0.94 | 1.19 | 1.48 | 0.83 | 0.55 | 0.55 | 0.56 |
| **A1.R3469F** | 0.97 | 1.34 | 1.08 | 1.12 | 0.42 | 1.37 | 1.37 | 1.13 | 1.31 | 0.66 | 0.82 | 0.71 | 0.94 |
| **A1/D.191947** | 0.84 | 1.19 | 1.54 | 1.34 | 1.10 | 1.14 | 1.07 | 1.19 | 1.60 | 0.95 | 0.82 | 0.87 | 0.72 |
| **NL** | 0.66 | 0.92 | 1.77 | 1.04 | 1.20 | 2.22 | 1.32 | 1.49 | 1.80 | 1.26 | 0.81 | 0.88 | 1.25 |
| **B.CH077** | 0.81 | 0.73 | 1.93 | 0.78 | 0.82 | 1.72 | 0.96 | 0.75 | 1.42 | 1.06 | 0.75 | 0.66 | 1.00 |
| **C.Z3618M** | 0.53 | 0.72 | 0.65 | 0.57 | 1.02 | 1.38 | 0.42 | 0.65 | 1.64 | 1.31 | 0.68 | 0.50 | 0.26 |
| **C.Z3678M** | 0.64 | 0.79 | 0.13 | 1.11 | 0.89 | 1.51 | 0.68 | 0.88 | 1.70 | 1.97 | 0.81 | 0.65 | 0.25 |
| **C.ZM247F_V2** | 1.07 | 0.94 | 2.08 | 1.22 | 1.27 | 1.51 | 1.20 | 0.96 | 1.63 | 1.03 | 0.97 | 0.96 | 0.53 |
| **C.Z1123M** | 0.93 | 0.62 | 0.87 | 0.56 | 0.73 | 1.47 | 0.64 | 1.50 | 1.37 | 0.50 | 0.76 | 0.47 | 0.90 |
| **D.191882** | 0.79 | 0.93 | 0.41 | 1.13 | 0.85 | 1.82 | 0.55 | 0.50 | 1.54 | 1.29 | 1.08 | 0.80 | 0.53 |

**S3 table. CD8 T-cell mediated inhibition of HIV-1 replication.** Inhibition was determined by the log_10_ reduction in relative light units of cultures of CD4 T-cells infected with one of ten luciferase gene engineered HIV-1 infectious molecular clones (IMC) and co-cultured with autologous CD8 T-cells compared with cultures of HIV-1 infected CD4 T-cells cultured alone.
