# Supplementary material for: Breadth of CD8 T-cell mediated inhibition of replication of diverse HIV-1 transmitted-founder isolates correlates with the breadth of recognition within a comprehensive HIV-1 Gag, Nef, Env and Pol potential T-cell epitope (PTE) peptide set
Source: PLoS One. 2021 Nov 17;16(11):e0260118. doi: 10.1371/journal.pone.0260118 (PMC8598018; doi:10.1371/journal.pone.0260118)
Supplement: Attachment — Submitted filename: Response to reviewers PONE-D-21-25896.pdf [file pone.0260118.s007.pdf]

IAVI Human Immunology Laboratory
Imperial College London
Chelsea & Westminster Hospital
369 Fulham Road, London, UK
Tel: +44 (0) 20 33157869

p.hayes@imperial.ac.uk
www.imperial.ac.uk

Dr. Peter Hayes
Senior Scientist

Imperial College London

21st October 2021

PONE-D-21-25896: Breadth of CD8 T-cell mediated inhibition of replication of diverse HIV-1 transmitted-founder isolates correlates with the breadth of recognition within a comprehensive HIV-1 Gag, Nef, Env and Pol potential T-cell epitope (PTE) peptide set

Dear Professor Nixon,

I thank you and the PLOS ONE team for the recent positive review of the above titled manuscript. I accept your invitation to submit a revised version addressing the points raised during the review process. Marked and unmarked versions of the manuscript will be uploaded.

I respond to the points raised in order as follows:-

Journal requirements

1) I have checked and have ensured that the manuscript meets PLOS ONE's style and file naming requirements.
2) I have revised the funding information:-

This work was made possible by IAVI, which was supported by funding from many donors, including the generous support of the American people through the US Agency for International Development award number AID-OAA-A-16-00032, the Bill and Melinda Gates Foundation, the Ministry of Foreign Affairs of Denmark, Irish Aid, the Ministry of Finance of Japan in partnership with The World Bank, the Ministry of Foreign Affairs of the Netherlands, the Norwegian Agency for Development Cooperation and the United Kingdom Department for International Development. The full list of IAVI donors is available at: http://www.iavi.org. The funding donors had no role in the study design, data collection and analysis, decision to publish, or preparation of the manuscript.

3) I confirm the ethics statement only appears in the Methods section of the manuscript.
4) The list of IAVI protocol C investigators and contact details are provided in the acknowledgements section. There is not one single lead author but the group can be contacted via Matt A. Price (email MPrice@iavi.org).

   The reference list has been reviewed, remains unchanged and none of the cited papers have been retracted.

Reviewer's comments

In comments 1 to 4, the reviewer stated the manuscript complies with the requirements.

Comment 5: I thank the reviewer for highlighting the need to use less stigmatizing vocabulary. I have reviewed the information provided and have revised the manuscript accordingly. "HIV-1 infected subjects" and similar terminology has been replaced with "subjects living with HIV". "HIV-1 infection" has been replaced with "acquisition of HIV". Such changes can be reviewed in the marked-up version of the revised manuscript. I have also alerted my immediate

colleagues to this matter and will inform the manuscript co-authors upon formal acceptance for publication. I will commit to using more appropriate vocabulary in future manuscripts, data presentations and communications.

Page 4 line 103 (original and revised unmarked manuscript): the typo “where” has been replaced with “were”.

Page 5 line 112 (original and revised unmarked manuscript): the selection process was not completely random and the criteria for selection have now been included. The priority was to recruit healthy subjects living with HIV, who were anti-retroviral naïve and willing to donate an extra blood sample for this study, which would not be completely random.

Page 9 line 195 (now line 199 of revised unmarked manuscript) and page 20 fig 2: The CMV and HIV peptides and other stimuli were not added together. To make this clearer the text has been modified to: “…. with one of the following stimuli;…” and fig 2 states “CMVpp65 or HIV-1 Nef”. The peptide concentration is re-stated on page 9 line 201 of the revised unmarked manuscript.

Page 20 line 395 (original manuscript): The term “spot forming units” and abbreviation (SFU) was stated on page 8 line 174 of the original and revised unmarked manuscript.

Fig 4: The typo testred has been corrected to tested.

Comment 6: I note the reviewer has elected to reveal their identity and that PLOS authors have the option to publish the peer review history. I agree to this option.

I have uploaded the original figure files to PACE. The original files met with PACE requirements but in reviewing the pdf previews I have decided to reduce the size of figures 2, 3 and 5 so as not to take up a full page for each figure. The revised figures have also been uploaded to PACE without messages indicating issues under “image problems” or “PACE adjustments”.

I thank the reviewer, editor and PLOS ONE team for their efforts and consideration of the manuscript for publication.

Yours sincerely,

Peter Hayes

Digitally signed by Peter Hayes
Date: 2021.10.21 13:04:33 +01'00'

Peter Hayes

Imperial College London
